# Experimental evaluation of insecticidal paints against *Triatoma infestans *(Hemiptera: Reduviidae), under natural climatic conditions

**DOI:** 10.1186/1756-3305-2-30

**Published:** 2009-07-08

**Authors:** Ivana Amelotti, Silvia S Catalá, David E Gorla

**Affiliations:** 1 Centro Regional de Investigaciones Científicas y Transferencia Tecnológica de La Rioja (CRILAR). Entre Ríos y Mendoza s/n (5301) Anillaco, La Rioja (Argentina)

## Abstract

**Background:**

*Triatoma infestans *is the main vector of Chagas disease in the Gran Chaco region of South America. The traditional spraying technique used for the application of pyrethroid insecticides has shown low efficiency in the elimination of the vector species populations occupying peridomestic structures of rural houses in the endemic area of Argentina. As part of studies looking for better alternatives, we evaluated the residual effect of insecticidal paints on the mortality of fourth instar nymphs of *T. infestans*.

**Results:**

The study was based on an experimental design that included two groups treated with an organophosphate (Inesfly 5A IGR™) and a pyrethroid (Inesfly 5A IGR NG™) formulations of the paint, that were applied on wood, cement blocks and adobe bricks under natural climatic conditions. A third group was an untreated control. Both paint formulations showed very long residual activity, producing mortality of 84% and 98% (pyrethroid and organophosphate formulations, respectively) after 12 months of the paint application. After eight months, nymphs exposed during 6 hours to the painted surfaces with the pyrethroid and organophosphate formulations showed 81.33% and 100% mortality, respectively.

**Conclusion:**

The organophosphate- and pyrethroid-based insecticidal paints showed a very long residual activity on the mortality of fourth instar nymphs of *T infestans*, compared with the traditional spraying technique used for the application of pyrethroid insecticides in peridomestic structures of rural houses in the endemic region for Chagas disease in the Gran Chaco of Argentina. The application of the paints by trained personnel of the vector control programmes could be considered as an alternative control tool in areas where the traditional methods have failed or showed low efficacy.

## Background

Chagas disease or American trypanosomiasis, is an infectious disease caused by *Trypanosoma cruzi*, transmitted by hematophagous insects of the subfamily Triatominae (Reduviidae). The disease is a major public health concern in Latin America, with estimates of 50000 people dying each year, 10 million people being infected, and around 100 million people being reportedly at risk of infection [1]. Although the disease is endemic in Latin American countries, human migration has spread the disease to other continents, especially North America and Europe [2,3]. In spite of long-term research efforts, there is no available vaccine [4] and treatment after infection is effective only for recent infections [5-7]. The preventive approach through the elimination of domestic populations of the insect vectors and systematic screening in blood banks is considered the best strategy to decrease the impact of the disease [8].

*Triatoma infestans *is the main vector of Chagas disease in Latin America. In the southern cone countries of South America, the control of the domestic populations of the species has been successfully achieved through national and international initiatives in more than 80% of the region originally occupied by *T infestans *[9,10]. The pyrethroid insecticides have shown high efficacy in the elimination of intradomestic populations of Triatominae. Unfortunately, the same pyrethroids have shown lower efficacy when applied to peridomestic populations [11,12]. A number of studies have been carried out on different vector control techniques to overcome the problem of the low efficacy of the pyrethroids in the peridomiciles. Cécere et al [13] reported that a duplication of the standard concentration of pyrethroid (25 mg a.i./m^2 ^of deltamethrin or equivalent) showed better performance when applied to goat corrals in the Argentine Chaco. Reithinger et al [14,15] reported long lasting effects on *T infestans *when using deltamethrin treated collars on dogs. Fipronil, formulated as pour-on on dogs, has controversial reports as a tool for the control of *T infestans *in peridomestic structures [16,17], whereas Amelotti et al [18], found high mortality and a decrease of blood intake in *T infestans *fed on chickens treated with cypermethrin pour-on. Earlier studies reported that β-cypermethrin impregnated fabrics kept dwellings free of *T infestans *[19], and deltamethrin impregnated curtains were considered as good options for the control of *Rhodnius *spp in Venezuela [20]. Fumigant canisters based on β-cypermethrin were developed and used during the 1980s [21], and fenitrothion has been used in northern Argentina where *T infestans *was found resistant to pyrethroids [22]. Insecticide suspension with vinyl polymers containing malathion were studied as early alternatives for Triatominae control by Oliveira Filho [23]. Studies developed under the sponsorship of WHO/TDR have reported an efficient control of Triatominae when these polymers were used indoors and in the peridomestic structures [24].

The objective of this work was to study the efficacy and the residual effect of two insecticide paints (Inesfly 5A IGR™ and Inesfly 5A IGR NG™) on *T infestans*, under experimental conditions, measuring residual activity on nymph mortality rate on different surfaces (wood, cement block and adobe bricks).

## Methods

The study was carried out under natural climatic conditions, between March 2008 and April 2009 at the facilities of CRILAR (La Rioja, Argentina), located at 28° 48' 44" S latitude and 66° 56' 14" W longitude. The paints were applied on 3 different materials, commonly used in rural house buildings. The mortality of fourth instar nymphs of *T infestans *was tested as a response variable.

### Insecticidal paints

The insecticidal paints were Inesfly 5A IGR (OP formulation) containing diazinon (1.5%), chlorpyrifos (1.5%) and pyriproxyfen (0,063%) (insect growth regulator, IGR) as active ingredients (OP formulation), and Inesfly 5A IGR NG containing α-cypermethrin (0.7%), d-alethrin (1%) and pyriproxifen (0,063%) as active ingredients (P formulation). Both formulations are vinyl paints with an aqueous base, with the active ingredients residing within Ca CO_3 _+ resin microcapsules. The formulation allows the gradual release of the active ingredients, increasing persistence and reducing the hazard to other organisms. The microcapsules have a morphological structure composed by an active nucleus, surrounded by a thin frame polymer that encloses the active ingredients and pigments. The product is a suspension of microcapsules that range from one to several hundred micrometers in size. Successful results of this paint formulation have been reported for the control of mosquitoes (Mosqueira et al, unpublished results) and cockroaches (*Periplaneta americana*) (Lopez et al, unpublished results) and *T infestans *[25].

### Insects

Insects used in this study were unfed fourth instar nymphs of *T infestans*, provided by the breeding facility of the Coordinación Nacional de Control de Vectores at Punilla (Córdoba). The specimens were F1 descendants of *T infestans *collected north of the province of San Luis (Argentina), bred under controlled conditions and fed on chickens (*Gallus *sp). The nymphs were used on average 7 days after moulting, and were kept under controlled temperature (26–28°C) and humidity (50–70% RH) in appropriately labelled plastic jars.

### Experimental design

Nine experimental groups (3 treatments × 3 surfaces) were included in the experimental design. One experimental group contained surfaces treated with the P formulation, a second group had surfaces treated with the OP formulation and a third group was included as control, with untreated surfaces. The paint was diluted in water (50%) and manually laid in two layers with brushes on 3 types of surfaces (adobe, wood and cement blocks). Each surface material was 15 × 15 cm. Paint was applied and left to dry for 48 hours. The 90 pieces of surface materials were placed under a straw roof without walls that avoided direct midday sunlight and rain. A plastic ring, 10 cm in diameter and 8 cm high maintained the nymph contact with the treated surface. Each experimental group had 10 replicates (except day 240, see below). On each replicate, 10 *T infestans *fourth instar nymphs were exposed to the surfaces during 7 days at 1, 47, 80, 125, 180, 240 and 365 days after paint application. The recommended exposure time for insecticide evaluation on Triatominae by WHO [26] is 24 or 48 hours, although a variety of exposure times have been used by different authors, from a few hours to several days [26-29]. In this case, we decided to expose the insects during 7 days because of the action mode (slow release formulation) of the insecticidal paint. Considering the importance of obtaining comparable measurements with other products, we carried out an evaluation of the effect of the exposure time (from 2 hs to 7 days) after 240 days of the paint application (see below). The active ingredient dosage on the assays was estimated from weight of the paint layer applied to ceramic tiles. Six tiles were individually weighed before and after painted in the same way the experimental surface materials were painted (50% dilution, two layers manually applied with brush). Average temperatures during the 7 day exposure periods are shown in Table 1.

**Table 1:** Average, minimum and maximum temperatures (°C) during the 7-day experimental periods.

Days after paint application	Average temperature	Minimum Temperature	Maximum Temperature
1	18.4	15.4	28.0
47	17.8	9.7	30.4
80	5.35	-2.9	22.4
125	12.6	4.4	25.2
180	13.9	3.1	23.9
240	21.8	13.3	31.6
365	19.5	12.2	28.1

After the exposure period, the nymphs were kept in appropriately labelled plastic jars with folded paper under the above mentioned controlled conditions during another 7 days to allow for eventual recovery of knocked down individuals that received sub-lethal pyrethroid concentration [30].

On day 240 of the paint application, the effect of exposure time to the treated surfaces on the mortality of nymphs was studied. Each of the 9 experimental groups had 5 replicates. On each replicate, groups of 5 fourth instar nymphs were exposed to the surface during 2, 6, 24, 48 and 168 hours. After the exposure during the specified time, the nymphs were kept in appropriately plastic jars under the above mentioned controlled conditions during 15 days, when mortality was recorded.

Data were analysed using parametric ANOVA, were variance heterogeneity was rejected (Levene test). Cases with heterogeneous variances were analysed with the Kruskal-Wallis test. All statistical calculations were carried out with Statistica [31].

## Results

### Residual effects

Under the experimental conditions, the dosage (per m^2^) of the OP formulation was 254 mg for diazinon, 254 mg for chlorpyrifos, and 11 mg of pyriproxifen. The dosage of the P formulation was 248 mg for alphacypermethrin, 239 mg for d-alethrin and 22 mg for pyriproxifen.

The mortality of the nymphs exposed on day 1 was 100% in all treated surfaces, with both formulations. After 47 days, nymph mortality of the treated groups was higher than 96% in both formulations and all surfaces. At day 80, nymph mortality in the pyrethroid group decreased to 73% on cement block, 74% on adobe and 80% on wood, significantly lower than the nymph mortality of the organophosphate group, which maintained 100% mortality (p < 0.001). The drop of the mortality in the P formulation coincided with very low winter temperatures, that was around 0°C during the exposure period and strongly restrained nymphs motility. After 125 days, the nymphs exposed to the two paint formulations showed mortality rates higher than 95%. At day 180 the mortality rate was higher than 88% except in the cement blocks treated with the P formulation, that was significantly lower than in the other experimental groups (74% ± 12.65, (p < 0.001) (Table 2). After 240 days of the paint application, overall mortality (all surfaces) of nymphs in both treatment groups (76–96% for the P formulation, 100% for the OP formulation) showed highly significant differences with the mortality of the control group (2.07% ± 1.72) (p < 0.001). No difference between the recorded mortality of the nymphs exposed to the OP and P formulations was found in wood and adobe surfaces (higher than 88%, p > 0.05). Significantly lower (p < 0.01) mortality was recorded in the nymphs exposed to the P formulation applied on cement blocks (76% ± 32.86 P formulation vs 100% OP formulation). After 365 days the nymphs exposed to the OP formulation maintained mortality rates higher than 97%, whereas the ones exposed to the P formulation were significantly lower, with mortality rates between 82 and 86%. Both formulations showed significant differences from the control group (Table 2). The OP formulation showed no decrease in the mortality produced in the fourth instar nymphs between day 1 and 365 (100% and 99%, respectively, p > 0.05). The P formulation showed a small but significant decrease in the mortality produced in the fourth instar nymphs between day 1 and 365 (100% and 83%, respectively, p < 0.05). After 365 days, both paint formulations maintained a complete coverage of all treated materials (wood, adobe and cement). The white colour of the paint was well maintained and adhered to the surfaces, even on adobe bricks.

**Table 2:** Mortality of nymphs of *T. infestans *exposed to insecticidal paints applied to three material surfaces.

			Mortality (%) in each experimental group Average (standard deviation)		
Days after paint application	Substrate	n	OP formulation	Pyrethroid formulation	Control

1	Cement	10	100(0)a	100(0)a	0(0)b
	Wood	10	100(0)a	100(0)a	1(3.16)b
	Adobe	10	100(0)a	100(0)a	1(3.16)b

47	Cement	10	100(0)a	96(5.16)a	1(3.16)b
	Wood	10	100(0)a	98(4.22)a	4(6.99)b
	Adobe	10	100(0)a	99(3.16)a	1(3.16)b

80	Cement	10	100(0)a	73(15.67)b	1(3.16)c
	Wood	10	100(0)a	80(17)b	2(4.22)c
	Adobe	10	100(0)a	74(21.7)b	0(0)c

125	Cement	10	100(0)a	95(5.27)b	5(9.72)c
	Wood	10	100(0)a	96(6.99)ab	0(0)d
	Adobe	10	100(0)a	95(7.07)b	0(0)d

180	Cement	10	100(0)a	74(12.65)b	4(4.14)d
	Wood	10	100(0)a	88(12.29)c	4(2.78)d
	Adobe	10	98(6.32)a	89(14.5)c	3(4.83)d

240	Cement	5	100(0)a	76(32.86)b	8(10.95)c
	Wood	5	100(0)a	88(10.95)ab	4(8.94)c
	Adobe	5	100(0)a	96(8.94)a	0(0)d

365	Cement	10	97(3)a	83(3.7)b	7(3)c
	Wood	10	100(0)a	86(3)b	6(2.2)c
	Adobe	10	100(0)a	82(2.9)b	5(3.1)c

OP: organophosphate formulation; P pyrethroid formulation; N: number of replicates with fourth instar nymphs. Same letters denote homogeneous groups within the same time after paint application P > 0.05, Factorial ANOVA.

Possible IGR effects on moulting were not measured, because of the small number of surviving individuals on both treatments.

### Effect of exposure time after 240 days

**Figure 1:**
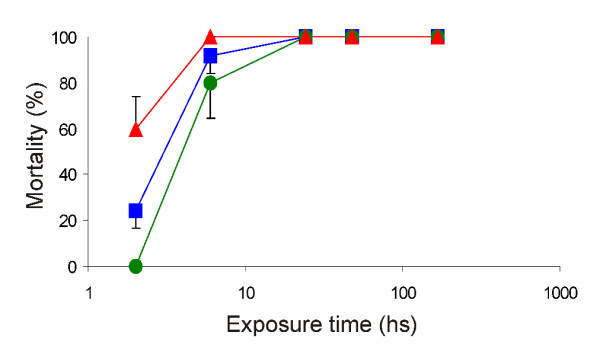
***T. infestans *mortality rate exposed on organophosphate formulation**. Mortality rate of fourth instar nymphs of *T. infestans *exposed during different times on three surfaces treated with the organophosphate formulation of the tested paint [adobe ■ (black square); wood ▲ (black triangle); cement ● (black circle)]. Vertical lines are standard error.

**Figure 2:**
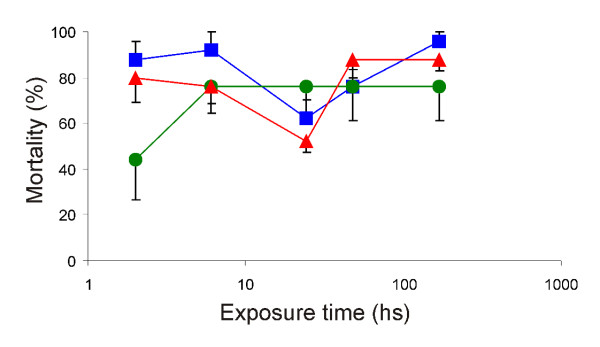
***T. infestans *mortality rate exposed on pyrethroid formulation**. Mortality rate of fourth instar nymphs of *T. infestans *exposed during different times on three surfaces treated with the pyrethroid formulation of the tested paint [adobe ■ (black square); wood ▲ (black triangle); cement ● (black circle)]. Vertical lines are standard error.

The study of the exposure time effect of the nymphs to the formulations showed that the OP formulation was slower acting than the P formulation to produce the highest value of nymph mortality. The mortality produced by the OP formulation increased with the exposure time, from 0% mortality after 2 hour exposure in cement to 100% after 24 h in all surfaces. Mortality increased at a lower rate when the exposure was on cement, and faster when exposure was on wood (Figure 1). The P formulation produced 45% mortality on cement block after a 2 hour exposure. Significantly higher mortality (> 80%, p < 0.05) was produced on wood and adobe after 2 hours of exposition (Figure 2). During the first 2 hours, the P formulation on cement and adobe showed higher mortality than the OP formulation (P < 0.004 in both cases). After a 6 hour exposure there were no differences in the effect on mortality among the OP and P formulations, in none of the surfaces considered. All treated groups showed higher mortality rate than in the control groups at all exposure times evaluated (p < 0.05).

## Discussion

This study showed that the insecticidal paints Inesfly 5A IGR and Inesfly 5A IGR NG, have a long term effect on the mortality of fourth instar nymphs of *T infestans*. After 240 days, a 6 hour-exposure to the organophosphate- based insecticidal paint caused 100% mortality, whereas the pyrethroid-based insecticidal paint caused mortality higher than 76%, when applied to either surfaces (cement, wood or adobe). The residual activity in these bioassays was longer than the majority of the insecticide formulations so far tested against vectors of Chagas disease in America, that typically show a 6 month residual activity, and a much longer effect than the reported for deltamethrin applied on similar surfaces frequently found in the Gran Chaco of Argentina [10,29,32]. The dosage of the OP active ingredients in the studied paint is 25% the amount recommended for other OPs (as fenithrotion and malathion)[33]. The dosage of the alphacypermethrin in the studied paint is 4.1 times higher than the recommended dosage (60 mg/m^2^) [34], although the effective dose on the paint surface is lower, because of the slow release mechanism that constitutes the originality of this paint.

This study also showed that the effect of the OP formulation was longer lasting than the P formulation after 365 days of paint application in all the studied surfaces. After 180 and 240 days, the cement blocks treated with the P formulation produced lower mortality rates than the other surfaces treated with the same insecticide. This finding is different to the effects normally reported on pyrethroids applied on different surfaces [32], where cement is normally reported as the surface where the insecticide shows better performance and wood, the worse [10]. This change in insecticide performance on different surfaces seems to be associated with the physical behaviour of the paint, which tends to fill the surface porosity, provided pores are relatively small, as in wood or adobe. When pores are bigger, as in un-smoothed cement, the amount of insecticide in contact with the triatomines seems to be smaller.

A number of studies showed that pyrethroids are less active at high temperature [35]. In this study, an unplanned record of the pyrethroid effect under low temperature (near 0°C during one week) showed that the pyrethroid had a lower effect on mortality presumably because of the motility decrease of the nymphs.

The results of this bioassay are coincident with a preliminary evaluation of the same OP formulation applied to houses with high infestation by *T infestans *in a region of the Bolivian Chaco where these paints were applied by compression sprayers [25]. The authors reported that the paint had good handling characteristics, gives a good appearance to houses, and that its acceptance among the population and the local sanitary authorities is excellent, contrasting with past experiences of OP-based insecticidal paints [24,36].

The toxicological issue of the OP formulation is an important factor to be considered, but it is outside the scope of this study. However, it is worth noting that a similar slow release emulsifiable suspension (SRES), using 5% formulation of malathion as an active ingredient used for the control of kala-azar vector in India, showed that cholinesterase levels of spray men and local dwellers remained at normal levels one week, one month and one year after the SRES application. Lal et al [37] concluded that the malathion formulation may be indicated as safe for use as a vector control measure and can be safely applied in the endemic area of kala-azar in Bihar so long as there is good personal protection for the spraymen during application. Additionally, control programmes of other insect vectors make widespread use of OP formulations, as is the case of the indoor residual spraying for malaria control [38]

One of the unsolved problems for *T infestans *control in the Chaco region is the low efficacy of pyrethroid spraying applied on peridomestic structures, resulting in a high resilience of the *T infestans *populations [39]. This paint formulation could be considered as a new alternative control tool for the triatomine populations, especially in the peridomestic structures, where the traditional application of emulsifiable concentrates of pyrethroid have failed or showed low efficacy. Contrasting with the SRES formulations tested during the 1980s, the studied formulations have the added value of house embellishment, offering a better aspect to the walls that might improve the self esteem of the rural communities.

Provided health security regulations of the countries are met, the insecticidal paints are attractive alternative tools for the control of the population abundance of Triatominae, especially in peridomestic structures. A possibility that could be considered as a safety precaution is the application of the insecticidal paints by the personnel of the vector control programmes, using manual sprayers as used in Bolivia [25]

## Conclusion

The two studied formulations of insecticidal paints demonstrated a very long residual activity over fourth instar nymphs of *T infestans*. After 12 months of the paint application, the organophosphate formulation produced 100% nymph mortality, whereas the pyrethroid formulation produced an average nymph mortality of 84% on the three different surfaces studied (wood, cement and adobe). After 240 days of the paints application, the organophosphate formulation produced 100% nymph mortality after 6 hs of nymphs contact with the treated surfaces. At the same time, the pyrethroid formulation produced an average mortality of 87% after a contact time that varied from 2 hours to 7 days. In this case, mortality observed on adobe was higher than mortality observed on cement (96 ± 9% vs 76 ± 33%, P < 0.01). The insecticidal paints are control tools that could be considered as an alternative or a complement for the elimination of peridomestic populations of *T infestans*, where the traditional spraying technique of pyrethroid insecticides showed low efficacy.

## Abbreviations

gr: grams; mg: milligrams; a.i: active ingredient; m^2^: square meter; Ca CO_3_: calcium carbonate; °C: Celsius degree.

## Competing interests

The authors declare that they have no competing interests.

## Authors' contributions

All the authors have contributed substantially to this study.

IA carried out the assays performed the statistical analysis and drafted the manuscript. SSC participated in the design of the study and revised it critically for important intellectual content. DEG conceived the study, helped to draft the manuscript and participated in its design and coordination. All authors read and approved the final manuscript
